# Type 2 diabetes is associated with clinical progression of aortic stenosis: a nationwide retrospective study in Sweden

**DOI:** 10.1038/s41598-026-64970-2

**Published:** 2026-08-08

**Authors:** Silvana Kontogeorgos, Annika Rosengren, Oskar Angerås, Petur Petursson, Tatiana Zverkova Sandström, Michael Fu, Helen Sjöland, Martin Lindgren

**Affiliations:** 1 Department of Molecular and Clinical Medicine/Cardiology, Institute of Medicine, Sahlgrenska Academy, University of Gothenburg, Gothenburg, Sweden; 2 Department of Medicine, Geriatrics and Emergency Medicine, Region Västra Götaland, Sahlgrenska University Hospital/Östra Hospital, Diagnosvägen 11, 416 50 Gothenburg, Sweden; 3 Department of Clinical Physiology, Sahlgrenska University Hospital, Gothenburg, Sweden; 4 Department of Cardiology, Sahlgrenska University Hospital, Gothenburg, Sweden

**Keywords:** Aortic stenosis, Diabetes mellitus, Clinical progression, Cardiology, Diseases, Medical research, Risk factors

## Abstract

Patients with type 2 diabetes mellitus (T2DM) have elevated risk for aortic stenosis (AS). The impact of diabetes on prognosis and clinical progression in AS is not well documented. In this study we try to assess if patients with T2DM and AS have faster clinical progression compared to patients without diabetes. By linkage of nationwide Swedish registries, we identified 16,116 T2DM and AS individuals (cases) and compared them with 31,066 matched comparators (AS without diabetes) from general population concerning clinical progression, defined as progression from AS diagnosis to hospitalization for heart failure, aortic valve replacement, mortality, and composite outcome of these endpoints. Kaplan–Meier curves were used to compare outcomes between cases and comparators and Cox proportional hazards analyses to account for confounders. AS individuals (total 47,182, 16,116 cases and 31,066 comparators) had mean age 71 years. The cases were younger, men predominated (58% versus 53%) and had more comorbidities. During follow-up, cases had faster clinical progression- 11% higher hazard for valve replacement (95% CI 1.04–1.18), 14% higher hazard for heart failure hospitalization (95% CI 1.03–1.26) and 4% higher hazard for composite outcome (95% CI 1.01–1.08), but with no mortality excess (HR1.00, 95%CI 0.97–1.05). Preexisting T2DM seems to be linked to moderately more rapid clinical progression in AS.

**Supplementary Information:**

The online version contains supplementary material available at 10.1038/s41598-026-64970-2.

## Introduction

As global life expectancy and obesity rates rise, the prevalence of degenerative aortic stenosis and type 2 diabetes mellitus is increasing^1,2^, resulting in an expanding population of individuals with both conditions. Individuals with type 2 diabetes have an increased risk of developing aortic stenosis^1,2^, whereas patients with aortic stenosis have a higher prevalence of diabetes than the general population^3,4^.

Although diabetes is known to have a multitude of effects on the cardiovascular system, the impact of diabetes on the development and progression of aortic stenosis remains unclear. Type 2 diabetes has been associated with low-grade inflammation that may accelerate early valve changes^5^, and also influences the myocardium, resulting in left ventricular hypertrophy, microangiopathy, enhanced cardiomyocyte stiffness, and impaired diastolic function^6^. Dysglycaemia, even at prediabetic levels, was shown in two recent Swedish studies to be correlated with both aortic valve calcification^7^ and incident aortic stenosis^8^. Diabetes is also associated with poorer outcomes in patients who undergo valve surgery and is included as an aggravating factor in the American Society of Thoracic Surgeons (STS) score and in Euroscore II (in the latter only if treated with insulin).

Both conditions commonly have several co-morbidities, including hypertension, obesity and dyslipidemia^9,10^, all of which may also worsen prognosis.

While considerable advances have been made in the prevention and management of coronary heart disease and heart failure in people with and without diabetes, there is little improvement with respect to prevention or medical treatment for degenerative aortic stenosis. However, with the introduction of transcatheter aortic valve replacement (TAVR) procedures as an option to open-heart surgery an increasing number of older patients with multiple conditions are now eligible for intervention.

Existing studies^11–15^ have examined the impact of diabetes on prognosis in aortic stenosis, but most have included selected populations, such as asymptomatic patients monitored in valve clinics^11^, patients with severe stenosis only^15^ or limited to individuals who underwent aortic valve replacement^12–14,16^. As far as we know, no large-scale studies have examined whether incident aortic stenosis progresses differently in unselected patients with and without diabetes.

Monitoring the progression of degenerative aortic stenosis is essential to determine the need for aortic valve replacement, the only currently available treatment option*.* Progression can be defined as hemodynamic (using echocardiography), anatomic (using computer tomography), histopathologic, or clinical (towards death, incident heart failure or needing valve replacement). Whether diabetes induces more rapid progression has not been reliably established. Some studies showed that either diabetes or metabolic syndrome may accelerate the progression of aortic stenosis^4,17–20^, although one study concluded that diabetes did not affect progression^21^. These conflicting findings likely reflect heterogeneity in study cohorts, the definition or severity of the stenotic lesion, varying methods used to define progression or restricting analyses to patients with severe aortic stenosis only.

In this observational study our main objective was to assess if patients with type 2 diabetes progress faster and thus have faster clinical progression towards possible outcomes (aortic valve replacement, hospitalization for heart failure or death) compared to an unselected national cohort of patients with aortic stenosis.

## Methods

### Study population

The Swedish National Diabetes Register (NDR), established in 1996, aims to improve the quality of diabetes care in Sweden, and serves as a research resource. The NDR collects clinical, laboratory, and other relevant data gathered by physicians and nurses nationwide in outpatient and primary care settings. Patients are informed about the register, its scope and the option to decline participation. The NDR covers approximately 90% of patients with type 2 diabetes and 95% of those with type 1 diabetes in Sweden^22^. The criteria used to define diabetes are harmonized with the World Health Organizations criteria^23^.

Using data from the NDR and excluding patients with a diagnosis of aortic stenosis before 2001, along with a small proportion in which age and sex could not be confirmed, a cohort of 569,895 patients with type 2 diabetes was identified. The inclusion period spanned from 1 January 2001 to 21 December 2019. All patients diagnosed with incident aortic stenosis in the Swedish National Patient Register (NPR) according to the International Classification of Diseases 9th and 10th revisions (ICD-9 and ICD-10) codes regardless etiology during this timeframe were selected for the study. No echocardiographic data concerning aortic stenosis severity or aortic valve morphology were available in our study.

At first entry in the register, patients with type 2 diabetes were matched for age, sex, and county of residence with five controls without type 2 diabetes, randomly selected from the Swedish population register of all Swedish citizens, held by Statistics Sweden.

Comorbidity data were obtained by integrating data from the NDR with the NPR, which includes all hospital discharge diagnoses and outpatient specialist care since 1987. The ICD-9 and ICD-10 codes used in this study are presented in Supplementary Table 1.

For the cases, data on cardiovascular risk factors, laboratory data (creatinine, serum cholesterol, HbA1C, albuminuria) and antidiabetic treatment were available, while no such information existed for the matched controls. The missing data in NDR are shown in Supplementary Table 2. Information concerning the education level and income was obtained by linking the NDR with the Longitudinal Integration Database for Health Insurance and Labor Market Studies (LISA).

The study was approved by the Swedish Ethical Review Authority, Regional Ethics Review Board in Gothenburg (2019-02-28, 2019-01229, 2023-00521-02) and complies with the Declaration of Helsinki. Individual consent is not necessary for reporting patients to NDR or for their inclusion in studies such as ours.

### Outcomes

The outcomes studied were mortality (from all causes and cardiovascular disease, with the latter defined as the underlying cause of death as determined by any ICD-10 codes from I00-I99), hospitalization for heart failure (the first hospitalization with heart failure as main diagnosis) and aortic valve replacement, either by transcatheter aortic valve replacement (TAVR) or surgical aortic valve replacement (SAVR) and a composite of these outcomes, whichever occurred first. The Swedish Cause of Death Register provided mortality data (including date and cause of death), while data on heart failure hospitalizations and valve replacement procedures were identified from the NPR.

### Statistical analysis

The characteristics of the cases and controls are presented as frequencies (percentages) for dichotomous data, means (standard deviations) and medians with 25th and 75th percentiles (Q1; Q3) for continuous data. The differences between the study groups were assessed as follows: χ^2^ test of independence was used for categorical data; Student’s t-test was used to compare means; Wilcoxon signed rank test was also reported as, according to Kolmogorov–Smirnov test, none of the continuous variables were normally distributed. The cumulative incidence of all-cause mortality, cardiovascular mortality and composite outcome (all-cause, hospitalization for heart failure and aortic valve replacement) was evaluated using Kaplan Meier curves. The medians of the whole follow-up time and the time-to-outcome were tested by Log-Rank test. The cardiovascular mortality and aortic valve replacement were analyzed using cumulative incidence functions treating all-cause death as competing risk.

Cox proportional hazards analysis was performed to compare the risk difference between cases and controls regarding the separate outcomes. The first model was adjusted for sex, age, education and baseline comorbidities (atrial fibrillation, coronary heart disease, hypertension, obesity [as a recorded diagnosis], heart failure, renal failure, cancer, hyperparathyroidism, hyperlipidemia). The only variable used in adjustment was education and, to avoid loss of other data, we set missing Education level to own category. The second model was additionally adjusted for statins and antidiabetic drugs (oral antidiabetic drugs, insulin, and glucagon-like peptide-1 receptor agonists). The third model included additional adjustments to encompass all baseline treatments (antidiabetic drugs, angiotensin-converting enzyme inhibitors, angiotensin receptor blockers, beta-blockers, mineralocorticoid receptor antagonists, oral anticoagulation, diuretics, antiplatelet drugs). These variables were chosen considering their clinical meaningfulness. The proportional hazards assumption was assessed by including interaction terms between covariates and log of (follow-up time). When evidence of non-proportionality was observed, additional models including these time interactions were evaluated. Because these extended models yielded results similar to those of the primary Cox models, the conventional Cox models were retained for the main presentation. To avoid the loss of data, zero times were set to 1 h (0.000114079553 year).

The statistical analyses were performed using SAS software version 9.4 (SAS Institute Inc, Cary, NC, USA). The graphics were drawn using R version 4.2.2^24^. *P*-values < 0.05 were considered as statistically significant.

## Results

**Fig. 1:**
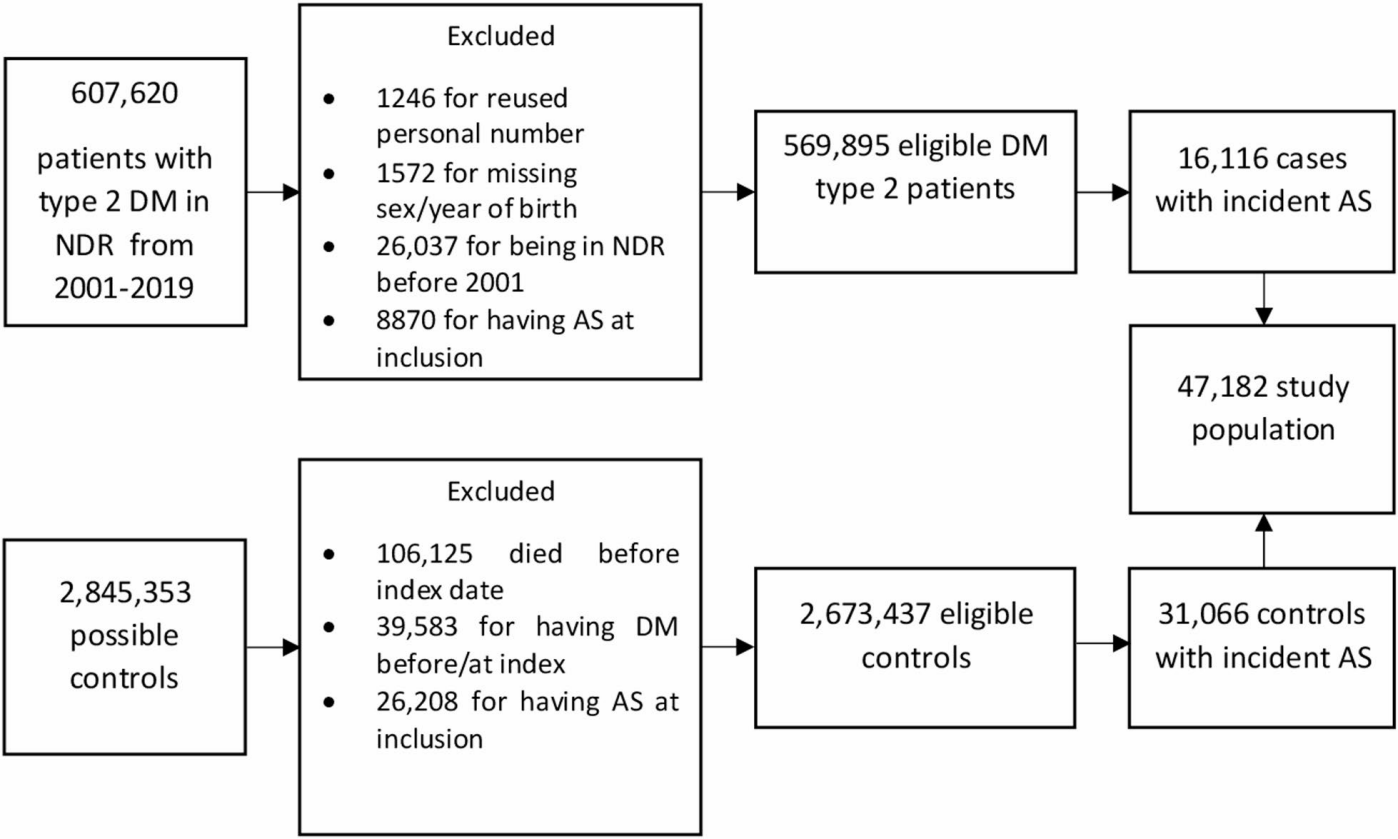
Flowchart of the study participants.

After excluding individuals with aortic stenosis at baseline and applying the criteria detailed in Fig. 1, we identified 16,116 individuals with incident aortic stenosis from a cohort of 569,895 individuals with type 2 diabetes, constituting the cases in our study. These 16,116 individuals were individually matched by sex, age and region with five non-diabetic comparators with aortic stenosis, randomly selected from the NPR. Of the 2,673,437 eligible controls, 31,066 had an incident diagnosis of aortic stenosis, resulting in a total of 47,182 individuals with aortic stenosis with and without diabetes in our study (Fig. 1).

The proportion of women in the diabetes group was significantly (*p* < 0.001) lower, 41.2%, compared to 47.1% in the comparator group. Patients with diabetes (the case group) were younger, with a mean age of 70.7 (SD 9.3) years compared to that of the comparators (72.0 years), (*p* < 0.001). In both groups, roughly 40% were aged 70–79 years, while the proportion of patients aged ≥ 80 years was higher among the comparators (22.5% vs 17.7%) (Table 1). Compared to the comparators, the group with diabetes had a marginally higher proportion of individuals with < 10 years of education and were more often born outside Sweden (14.3% vs 10.4%), and had lower income, (all *p* < 0.001). Individuals with diabetes had a greater prevalence of comorbidities at baseline compared to the comparator group (*p* < 0.001), valvular heart diseases, cardiomyopathies and haemorrhagic stroke were found in roughly the same proportion in the two groups, while cancer (*p* = 0.0257), endocarditis (*p* = 0.0138), congenital heart disease (*p* = 0.0161) and aortic aneurysm/dissection (*p* < 0.001) were diagnosed slightly more often in the comparator group.

**Table 1:** Baseline characteristics of patients diagnosed with aortic stenosis and type 2 diabetes (cases) compared with patients with aortic stenosis without diabetes (controls).

	DM cases	Controls	*p* value
Demographics			
N (%)	16,116	31,066	
Women, n (%)	6643 (41.2)	14,641 (47.1)	< 0.0001
Age, median (Q1, Q3)	71 (65;77)	73 (66;79)	< 0.0001
Age group:			< 0.0001
< 60	1934 (12.0)	3219 (10.4)	
60–69	4984 (30.9)	8282 (26.7)	
70–79	6354 (39.4)	12,579 (40.5)	
80 +	2844 (17.7)	6986 (22.5)	
Education (non-missing)			< 0.0001
< 10 years, n (%)	7649 (47.5)	13,866 (44.6)	
10–12 years, n (%)	5892 (36.6)	11,275 (36.3)	
> 12 years, n (%)	2259 (14.0)	5536 (17.9)	
Born outside Sweden, n (%)	2322 (14,3)	3240 (10,4)	< 0.0001
Married / cohabiting, n (%)	7957 (49.4)	14,941 (48.1)	0.0084
Income, (median)	1484	1510	< 0.0001
Follow-up time, (min, max)	0.0–18.1	0.0–18.9	
Follow-up time, median (Q1, Q3)	5.4 (5.3, 5.5)	6 (5.8, 6.1)	< 0.0001
Comorbidities			
Atrial fibrillation, n (%)	3234 (20.1)	5340 (17.2)	< 0.0001
Cardiomyopathy, n (%)	197 (1.2)	383 (1.2)	0.9220
Coronary heart disease, n (%)	4632 (28.7)	6018 (19.4)	< 0.0001
Myocardial infarction, n (%)	458 (2.8)	650 (2.1)	< 0.0001
Stroke any, n (%)	696 (4.3)	1041 (3.4)	< 0.0001
Ischemic stroke, n (%)	636 (3.9)	930 (3.0)	< 0.0001
Haemorrhagic stroke, n (%)	74 (0.5)	138 (0.4)	0.8178
Congenital heart disease, n (%)	68 (0.4)	184 (0.6)	0.0161
Heart failure, n (%)	3311 (20.5)	4999 (16.1)	< 0.0001
Hypertension, n (%)	8790 (54.5)	12,388 (39.9)	< 0.0001
Obesity, n (%)	1257 (7.8)	573 (1.8)	< 0.0001
Valvular disease, n (%)*	9528 (59.1)	18,475 (59.5)	0.4645
Deep venous thrombolism, n (%)	431 (2.7)	882 (2.8)	0.3022
Pulmonary embolism, n (%)	146 (0.9)	352 (1.1)	0.0220
Aortic aneurysm/dissection, n (%)	412 (2.6)	1358 (4.4)	< 0.0001
Cancer, n (%)	3783 (23.5)	7580 (24.4)	0.0257
Renal failure, n (%)	1443 (9.0)	1313 (4.2)	< 0.0001
COPD/asthma, n (%)	1473 (9.1)	2461 (7.9)	< 0.0001
Hypercholesterolemia, n (%)	836 (5.2)	1115 (3.6)	< 0.0001
Hyperlipidemia, n (%)	2827 (17.5)	2889 (9.3)	< 0.0001
Peripheral arterial disease, n (%)	1359 (8.4)	1515 (4.9)	< 0.0001
Endocarditis, n (%)	104 (0.6)	266 (0.9)	0.0138
Hyperparathyroidism, n (%)	753 (4.7)	1093 (3.5)	< 0.0001
Medication			
ACEi/ARB/ARNi, n (%)	3525 (21.9)	4823 (15.5)	< 0.0001
MRA, n (%)	458 (2.8)	1139 (3.7)	< 0.0001
SGLT2, n (%)	127 (0.8)	1 (0.0)	< 0.0001
Beta blockers, n (%)	513 (3.2)	740 (2.4)	< 0.0001
Diuretics, n (%)	6637 (41.2)	9144 (29.4)	< 0.0001
Anticoagulation, n (%)	2792 (17.3)	4258 (13.7)	< 0.0001
Antiplatelet drugs, n (%)	7450 (46.2)	10,253 (33.0)	< 0.0001
Statiner, n (%)	9012 (55.9)	9090 (29.3)	< 0.0001
Oral antidiabetics, n (%)	8451 (52.4)	309 (1.0)	< 0.0001
Glucagon-like peptide-1-receptor agonists, n (%)	270 (1.7)	5 (0.0)	< 0.0001
Insulin, n (%)	5032 (31.2)	254 (0.8)	< 0.0001
DM patients’ characteristics			
DM onset, mean (SD)	64.5 (11.3)		
DM duration (years), median (Q1, Q3)	3 (1, 9)		
Smoking, n (%)	1316 (8.2)		
Albuminuria, n (%)			
No albuminuria	6746 (41.9)		
Microalbuminuria (3–30 g albumin/mol creatinine)	1764 (11.0)		
Macroalbuminuria (> 3 0 g albumin/mol creatinine)	849 (5.3)		
GFR, (mL/min/1,73 m^2^), median (Q1, Q3)	80.09 (61.48, 89.93)		
Retinopaty, n (%)	1316 (8.2)		
Systolic BP, (mm Hg), median (Q1, Q3)	149 (130; 152)		
Diastolic BP, (mm Hg), median (Q1, Q3)	80 (70; 84)		
BMI, (kg/m^2^), median (Q1, Q3)	29.6 (26.7; 33.2)		
Serum creatinine, (μmol/l), median (Q1, Q3)	79 (67,93)		
HbA1C, (mmol/mol), median (Q1, Q3)	51 (45, 59)		
Total cholesterol, (mmol/l), median (Q1, Q3)	4.9 (4.2, 5.7)		
Physical activity, n (%)†			
Never	1269 (7.9)		
< 1 time/week	1188 (7.4)		
1–2 times/week	1773 (11.0)		
3–5 times/week	1619 (10.1)		
5 times/week	2525 (15.7)		

ACE,i angiotensin-converting enzyme inhibitor; ARNi, angiotensin receptor-neprilysin inhibitors, ARB, angiotensin receptor blockers; BMI body mass index; BP, blood pressure; COPD, chronic obstructive pulmonary disease; GFR, estimated glomerular filtration rate (estimated according to the Modification of Diet in Renal Disease Study Equation); HbA1C, glycated hemoglobin; MRA, mineralocorticoid receptor antagonists; SAVR, surgical aortic valve replacement; SD, standard deviation; SGLT2, sodium glucose co-transporter-2 inhibitors. The comparisons between groups are made using using the chi-squared, Student’s t-test or Wilcoxon signed rank test, as appropriate. *Other than AS, †Missing 48%

The mean duration of diabetes for individuals in the case group was close to 6 years, with a mean age of onset of 64 years. Of the cases, 8.0% were regular smokers, 8.2% had retinopathy, the mean blood pressure was 143/78 mm Hg, and the average BMI approached the threshold for obesity. The median value of HbA1C was 51 mmol/mol and 8845 individuals (55% of cases) had a good glycemic control considering a threshold of ≥ 53 mmol/mol indicating poor control. A large portion of the data on micro- and macroalbuminuria was missing (42%). Data on physical activity, defined as a period of pulse-increasing activity for at least 30 min per day, were missing in 48% of the cases. Among individuals with registered data, most were physically active, with 16% exercising at least five times per week. Patients with diabetes, compared to controls, were more likely to receive treatment with angiotensin-converting enzyme inhibitors (ACEi), angiotensin receptor blockers (ARBs) or angiotensin receptor-neprilysin inhibitors (ARNis), sodium-glucose co-transporter-2 inhibitors (SGLT2s), beta-blockers, diuretics, anticoagulation, antiplatelet and statins. The only drug class used more often in controls was mineralocorticoid receptor antagonists (MRAs). Insulin treatment was administered to 31% of individuals in the case group.

### Outcomes

**Fig. 2:**
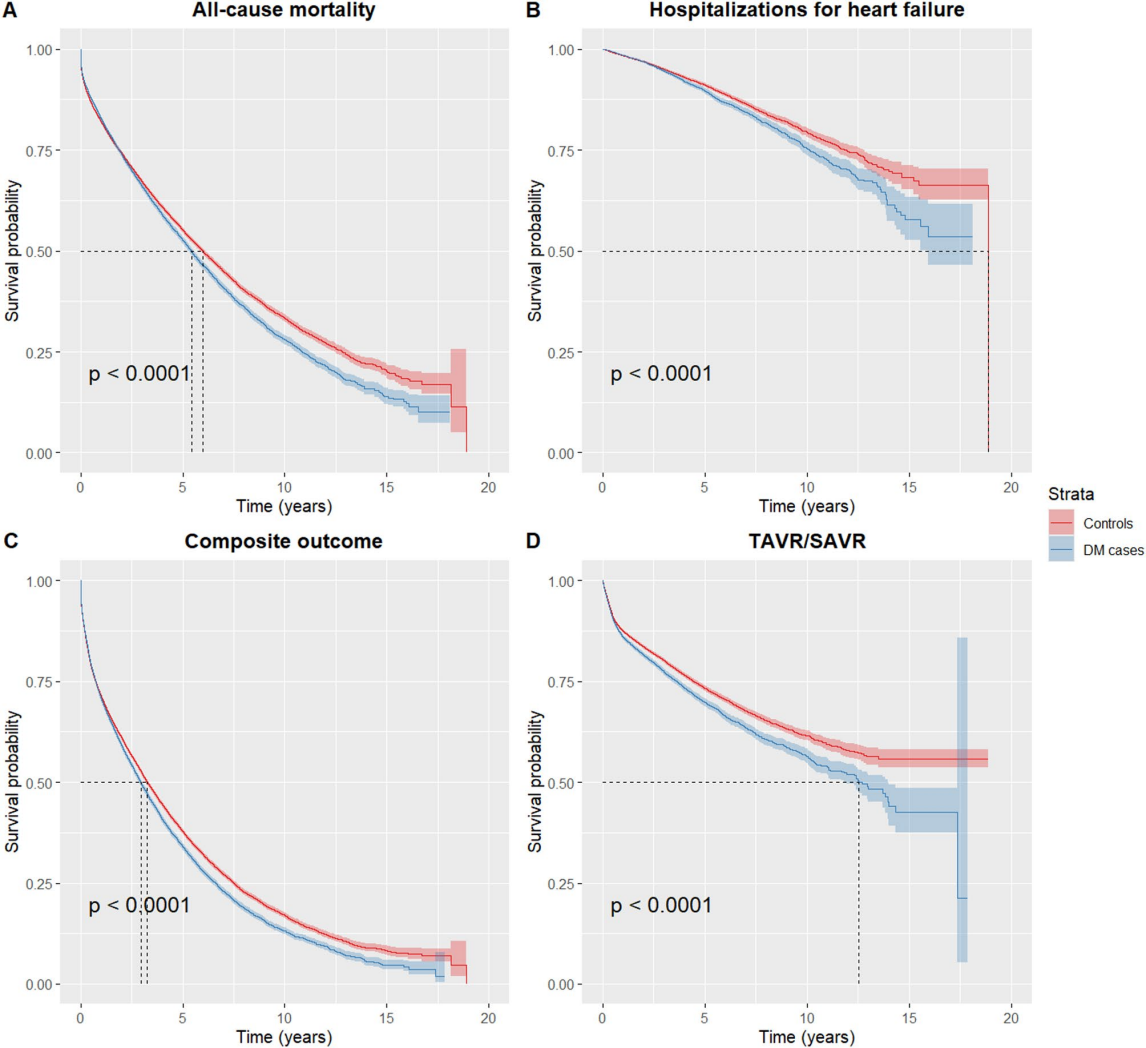
Kaplan–Meier survival curves illustrating outcomes for cases and controls.

Over a follow-up of up to 18 years, all outcomes for cases exceeded that of comparators with a high level of significance (*p* < 0.001 for all) (Table 2): Specifically, 48.2% of cases died, among them 27.2% from cardiovascular causes, while the corresponding death rates for comparators were 43.3% and 25.0% respectively. Patients with diabetes had more often been subject to TAVR/SAVR procedures (6.6% vs 5.1% for TAVR, 15.5% vs 14.1% for SAVR, incidence rate of 81.2 vs 70.1 per 1000 person years) and been hospitalized with incident heart failure (23.3 versus 20.0 per 1000 person years compared to comparators). The composite outcome (mortality, aortic valve replacement or heart failure hospitalization) was documented in 63.9% of cases and 58.5% of controls, corresponding to an incidence rate of 235.9 per 1000 person years in cases vs 215.9 per 1000 person years in comparators (Table 2). The progression towards death (regardless of underlying cause) and the composite outcome was moderately but significantly faster in patients with diabetes than in comparators. Survival free of the components of the composite outcome was higher in comparators (Fig. 2).

**Table 2:** Outcome incidence during follow-up, stratified by case group (aortic stenosis with type 2 diabetes) and control group (aortic stenosis without diabetes).

Outcomes	DM cases	Controls	*p*-value
All-cause mortality, n (%)	7769 (48.2)	13,456 (43.3)	< 0.001
All-cause mortality rate/1000 person-years	136.3	126.5	
Cardiovascular mortality, n (%)	4389 (27.2)	7778 (25.0)	< 0.001
Cardiovascular mortality rate/1000 person-years	77.0	73.1	
TAVR, n (%)	1069 (6.6)	1569 (5.1)	< 0.001
SAVR, n (%)	2505 (15.5)	4368 (14.1)	< 0.001
SAVR/TAVR rate/1000 person-years	81.2	70.1	
Hospitalizations for heart failure, n (%)	1328 (8.2)	2128 (6.8)	< 0.001
Heart failure hospitalizations rate/1000 person-years	23.3	20.0	
Composite outcome, n (%)	10,305 (63.9)	18,171 (58.5)	< 0.001
Composite outcome rate/1000 person-years	235.9	215.9	

Composite outcome consists of mortality, heart failure hospitalization or aortic valve replacement. HF, heart failure; HR, hazard ratio; SAVR, surgical aortic valve replacement; TAVR, transcatheter aortic valve replacement.

After adjusting for age, education and comorbidities (atrial fibrillation, hypertension, obesity, heart failure, renal failure, coronary heart disease, cancer, hyperparathyroidism, hyperlipidemia), patients with diabetes had a 10% higher hazard of death (95% CI 1.06–1.13), 32% higher hazard of hospitalization for heart failure (95% CI 1.23–1.42), 18% higher hazard of undergoing TAVR/SAVR (95% CI 1.13–1.23) and a 10% higher hazard of the composite outcome (95% CI 1.07–1.13), for all outcomes (*p* < 0.001). After further adjustment for medications (antidiabetic medications and statins in model 2, and further for all registered medications in model 3, not shown), no excess mortality risk was observed among cases compared to comparators. However, the disparity in hospitalization rates for heart failure, TAVR/SAVR and the composite outcome persisted, with cases having 14% higher hazard for hospitalization for heart failure (95%CI 1.03–1.26), 11% higher hazard for AVR (95% CI 1.04–1.18) and 4% higher hazard for the composite outcome (95%CI 1.01–1.08) (Table 3).

**Table 3:** Cox regression hazard models for all-cause, cardiovascular mortality and composite outcome in cases (type 2 diabetes and aortic stenosis) compared to matched controls (aortic stenosis without diabetes).

Outcomes	Model 1		Model 2	
	HR (95%CI)	*p* value	HR (95%CI)	*p* value
All-cause mortality	1.10 (1.06–1.13)	< 0.0001	1.00 (0.97–1.05)	0.8298
Cardiovascular mortality	1.11 (1.09–1.14)	< 0.0001	1.00 (0.95–1.05)	0.9867
Hospitalization for HF	1.32 (1.23–1.42)	< 0.0001	1.14 (1.03–1.26)	0.0085
TAVR/SAVR	1.18 (1.13–1.23)	< 0.0001	1.11 (1.04–1.18)	0.0009
Composite outcome	1.10 (1.07–1.13)	< 0.0001	1.04 (1.01–1.08)	0.0252

Model 1. Adjusted for sex, age, education, atrial fibrillation, coronary heart disease, hypertension, obesity, heart failure, renal failure, hyperparathyroidism, cancer and hyperlipidemia at baseline.

Model 2. Model 1 + statins and anti-DM treatment (oral antidiabetics drugs, insulin, Glucagon-like peptide-1 receptor agonist) at baseline.

HF, heart failure; HR, hazard ratio; SAVR, surgical aortic valve replacement; TAVR, transcatheter aortic valve replacement.

### Predictive factors from Cox proportional hazards analysis

**Fig. 3:**
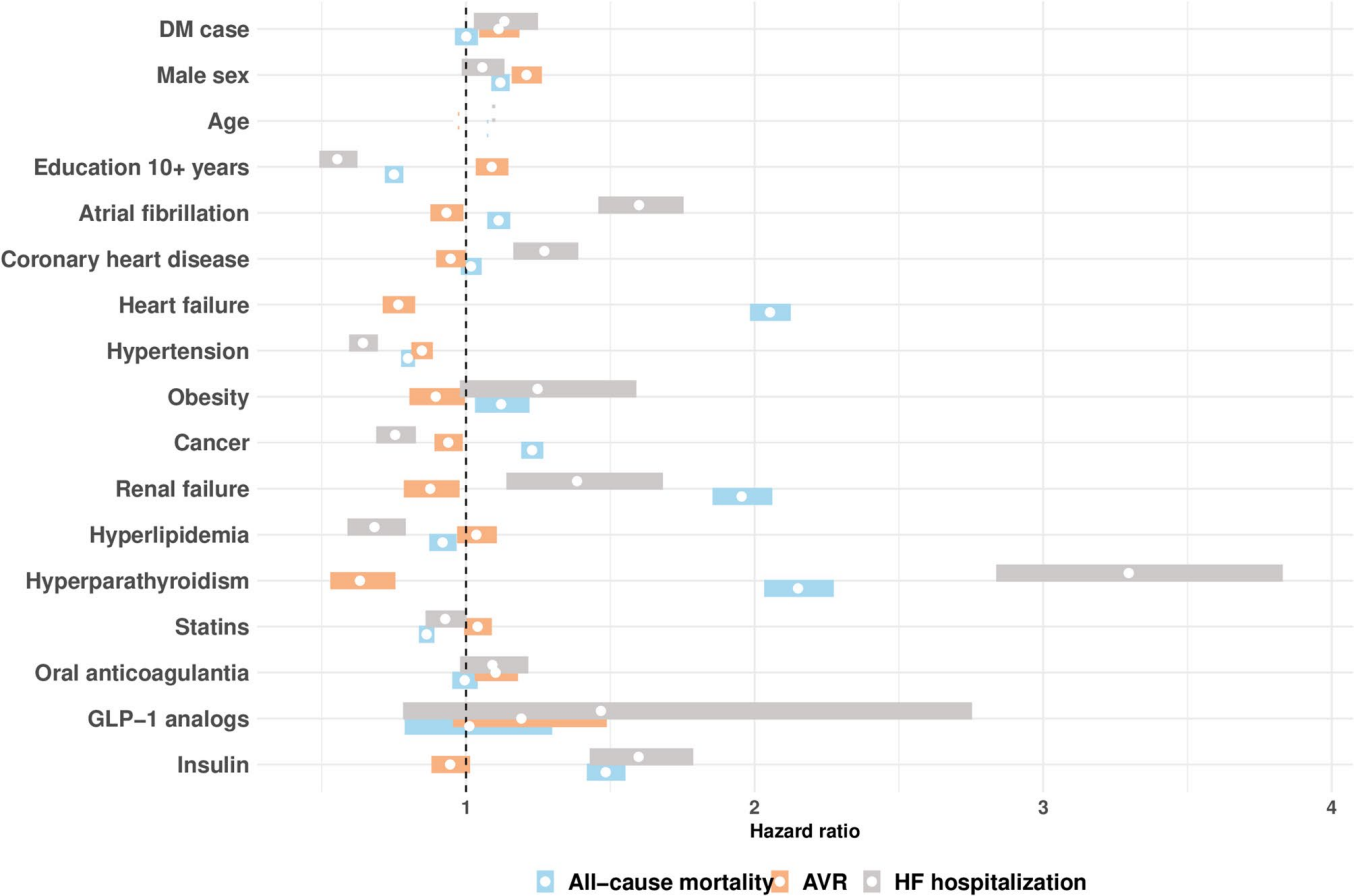
Forest plot of the factors influencing the constituents of the composite outcome in cases and controls.

Factors significantly associated with an elevated risk of outcomes after adjustment, including the composite outcome are presented in Fig. 3, showing that type 2 diabetes alone was a predictive factor for AVR (HR 1.11, 95%CI 1.04–1.18), heart failure hospitalization (HR 1.14, 95%CI 1.03–1.26), but not for all-cause mortality (HR 1.00 95%CI 0.96–1.05). Other factors correlated with higher all-cause mortality were male sex (HR 1.11, C95% 1.09–1.15), atrial fibrillation (HR 1.11, 95% CI 1.07–1.15), heart failure (HR 2.05, 95%CI 1.98–2.12), obesity (HR 1.12, 95% CI 1.03–1.22), cancer (HR 1.23, 95% CI 1.19–1.27), renal failure (HR 1.96, 95%CI 1.86–2.06) and hyperparathyroidism (HR 2.15, 95%CI 2.03–2-28) . Male sex (HR 1.21, 95%CI 1.16–1.26) and education over 10 years (HR 1.09, 95%CI 1.05–1.14) were associated with higher hazard for aortic valve replacement, while atrial fibrillation (HR 1.58, 95%CI 1.44–1.73), coronary heart disease (HR 1.27, 95% CI 1.17–1.39), renal failure (HR 1.41, 95% CI 1.16–1.71) and hyperparathyroidism (HR 3.24, 95%CI 2.79–3.77) were correlated with higher probability for hospitalization for heart failure.

The result of these factors on the composite outcome is presented in Supplementary Fig. 1, that shows that being male, having renal failure or hyperparathyroidism had a higher risk to reach the composite outcome compared to controls.

## Discussion

This nationwide retrospective cohort study of patients with aortic stenosis examined the impact of type 2 diabetes on mortality and a composite outcome encompassing death, hospitalization for heart failure, or TAVR/SAVR. Compared to individuals with aortic stenosis without type 2 diabetes, those with diabetes progressed faster towards death, had a significant but moderately higher risk of hospitalisation for heart failure and for undergoing aortic valve replacement. After adjusting for comorbidities, other potential risk factors and medications, no remaining association between diabetes and all-cause mortality persisted. However, the hazard for aortic valve replacement and hospitalization for heart failure remained elevated in individuals among patients with both aortic stenosis and diabetes.

Our results show that unadjusted mortality was higher in individuals with type 2 diabetes and aortic stenosis than in controls, but that this difference was attenuated after adjustment for comorbidities and medications. This finding should be interpreted cautiously and may reflect differences in baseline risk profile, treatment patterns, and residual confounding rather than a specific survival benefit of antidiabetic therapy.

Several studies found an association between diabetes and reduced survival in patients with aortic stenosis. However, these studies have either focused on patients undergoing TAVR^12^, SAVR^13,14^ or on asymptomatic patients with moderate or severe stenosis and preserved ejection fraction^11^. In the latter study^11^, 18% of participants had diabetes, which was associated with higher all-cause mortality, cardiovascular mortality and worse postoperative survival. A study from South Korea that included patients with diabetes and severe aortic stenosis, additionally using imaging and plasma proteomics analysis, concluded that diabetes was a predictive factor for heart failure and mortality in these patients, indicating that the extent of myocardial remodelling and diastolic dysfunction was more pronounced in individuals with diabetes^25^. Rosenhek et al. also found that diabetes constituted a negative prognostic factor for event-free survival (death or AVR) in 116 consecutive asymptomatic patients with very severe aortic stenosis^15^. Another recent study on 582 patients with severe aortic stenosis who underwent TAVR studied the influence of stress hyperglycemia ratio on all-cause mortality and composite outcome of mortality, readmissions for heart failure and major adverse cardiovascular events and found that stress hyperglycemia ration influences negatively all these outcomes^26^.

Even so, the association of diabetes with prognosis of aortic stenosis has, so far, not been sufficiently clarified. For example, a study in patients with moderately to severe aortic stenosis found no association between diabetes and cardiovascular events but observed that all-cause mortality was higher in individuals with diabetes^27^. Another study showed that the association between diabetes and survival in patients with aortic stenosis is dependent on the severity of the valvular stenosis, affecting cardiovascular mortality only in cases of severe aortic stenosis^28^. It is thus still unclear if diabetes affects survival in individuals with aortic stenosis overall.

Our current findings further indicate that patients with aortic stenosis and type 2 diabetes may experience a more accelerated clinical progression, from diagnosis to the possible clinical outcomes (mortality, valve replacement or heart failure requiring hospitalization) than comparators without diabetes. Several previous studies^17,18,29–31^ also indicate that diabetes accelerates the progression of aortic stenosis. Among them, one included patients from a long-term health care facility (thus a more selected cohort than ours)^17^, and another was a retrospective study of 166 consecutive patients (99% men) and examined the progression using echocardiography for diagnosis and serial echocardiography for follow-up. In both studies diabetes was correlated with hemodynamic progression of the stenotic lesion, but in the latter it led to a more rapid progression in individuals with moderately severe aortic stenosis, but not in those with heavily calcified valves^18^. Two additional investigations on explanted aortic valves obtained from patients with severe aortic stenosis following AVR indicated that diabetes accelerated progression at a histopathological level^29,30^. In another study on 276 patients with aortic stenosis examined with echocardiography age and diabetes were shown to be a factor that predicted rapid echocardiographic progression of aortic stenosis^32^.

Other studies have focused on the metabolic syndrome and its effect on aortic stenosis. For instance, a retrospective study from 2006 on 105 patients with at least moderate stenosis concluded that the presence of the metabolic syndrome increased the hemodynamic and clinical progression rate of aortic stenosis. However, an analysis focused only on diabetes revealed a tendency toward accelerated progression on gradients, while no such trend was observed in the dimension of the valvular area^19^. Another study, including participants in the Multi-Ethnic Study of Atherosclerosis (MESA) and using computed tomography for anatomical progression analysis, established a connection between diabetes, as a component of the metabolic syndrome, and an increased incidence of aortic valve calcification. This effect was observed in individuals with baseline calcification, although no influence on progression was detected^4^.

A few studies did not find an association between diabetes and progression. For example, a study of 203 patients with a mean age of 74 years concluded that diabetes did not influence aortic stenosis with respect to hemodynamic or anatomic progression (defined with echocardiography and computed tomography, respectively) during a mean follow-up of 3.2 years and a maximum of 6 years^21^. However, only 25% of the study participants had diabetes (type 2), and the authors acknowledged the potential for observing an effect with extended follow-up.

In our study individuals with aortic stenosis and type 2 diabetes had higher risk to be hospitalized for heart failure compared to controls. One study investigated the rehospitalization after TAVR on 750 patients between 2009 and 2017 and found that diabetes, both treated with insulin or oral antidiabetics was associated with increased risk of hospitalization for heart failure^33^, thus in line with our study. However, the higher risk of heart failure hospitalization in patients with type 2 diabetes could be interpreted as reflecting a more unfavourable clinical trajectory after aortic stenosis diagnosis, rather than accelerated valvular progression alone.”

Our study lacks information on aortic stenosis severity, impeding a comparative analysis of severity across the two populations (cases and controls). Despite comprehensive adjustments, including medication use, the risk associated with TAVR/SAVR intervention remained significantly higher in cases compared to controls. Because TAVR/SAVR indication is determined by symptoms and severity and not the presence of diabetes, the observed increased hazard may stem from more advanced valvular disease within the diabetic patient population.

We also found that some factors could lead to faster clinical progression of aortic stenosis in patients with type 2 diabetes. However, as these factors may exert distinct influence on the on the constituents of the composite outcome and our access to co-morbidity data is limited to baseline, we are unable to draw definitive conclusions about these findings.

### Strengths and limitations

The major strength of our study is the inclusion of a large cohort encompassing all Swedish individuals diagnosed with type 2 diabetes and aortic stenosis. Additional strengths lie in the extended follow-up period and the focus on a growing patient population with a combination of two common conditions. However, there are also some limitations, the most important of which is that we used diagnoses based on ICD codes without the possibility of validating them, lacking echocardiographic data on the initial severity of the valve lesion, which may have been more pronounced among patients with diabetes. A second important limitation is the absence of echocardiographic information regarding valve morphology (tricuspid or bicuspid), and the severity of the stenosis, with the latter a critical determinant in the progression, thus we could not distinguish degenerative tricuspid AS from bicuspid or other non-degenerative forms. However, taking into account the mean age of the individuals included (of about 70 years) most of the patients had most probably degenerative aortic stenosis. Still, a recorded diagnosis of aortic stenosis in different registers typically indicates at least moderate severity^34^ and even a moderate grade aortic stenosis seems to negatively influence the prognosis^35^. Possibly, individuals with diabetes have a more severe aortic stenosis at diagnosis, indicating a more advanced disease at a younger age. A third limitation concerns the lack of data on risk factors, clinical status, laboratory analyses, ECG or echocardiography for the control group and although we adjusted as broad as possible, there are parameters that could not be addressed in the adjustment of the analyses, including severity and duration of diabetes. In addition, because individuals were included between 2001 and 2019, glucose-lowering treatment patterns largely reflect an earlier therapeutic era, with limited use of SGLT2 inhibitors and GLP-1 receptor agonists, precluding robust analysis of these therapies in relation to outcomes. This likely also explains the relatively high proportion of insulin-treated patients and the comparatively modest use of oral glucose-lowering therapies in the diabetes group.”

A fourth limitation is that only hospital-based diagnoses were recorded, thereby excluding conditions treated and diagnosed solely by general practitioners, a group which may include very old and frail patients, who, nevertheless, might have benefited from valvular intervention. A fifth limitation is that individuals with type 2 diabetes may have more frequent healthcare contacts and may therefore be diagnosed with aortic stenosis earlier or under different clinical circumstances than individuals without diabetes, introducing potential surveillance and lead-time bias. Because echocardiographic severity at diagnosis was unavailable, we could not determine whether patients with diabetes entered follow-up at a more advanced stage of aortic stenosis. Accordingly, the observed differences in AVR and heart failure hospitalization may partly reflect differences in disease stage or timing of diagnosis rather than only faster progression after diagnosis. Nevertheless, the findings remain clinically relevant, as individuals with type 2 diabetes still had a less favourable clinical course after a recorded diagnosis of aortic stenosis.” In addition, in baseline table a small percentage of individuals (< 1%) had antidiabetic treatment despite not having diabetes. This could be explained by other indications (e.g. Polycystic Ovary Syndrome or temporary Insulin therapy) or by the latency between diagnosis and inclusion in the register. However, this small number of individuals would not affect the results.

## Conclusion

In this nationwide study, patients diagnosed with both aortic stenosis and diabetes seem to have a more rapid clinical progression compared to those with aortic stenosis only. After multiple adjustment including comorbidities and medications, it still appeared that diabetes contributed to an elevated risk for hospitalisation due to heart failure, AVR and the composite endpoint, which also included all-cause mortality. The clinical implications may include increased awareness concerning the unfavourable association between aortic stenosis and diabetes in planning the follow-up of these patients.

## Supplementary Information

Below is the link to the electronic supplementary material.

Supplementary Material 1.

Supplementary Material 2.

Supplementary Material 3.

## Data Availability

The individual-level data used for this study are available from the NDR, the Swedish National Board of Health and Welfare and Statistics Sweden, pending permission through the Swedish Ethical Review Authority and fulfilment of all other legal requirements. The data that support the findings of this study are available from the corresponding author upon reasonable request.

## Abbreviations

ACEi: Angiotensin converting enzyme inhibitors
ARB: Angiotensin receptor blockers
ARNi: Angiotensin receptor-neprilysin inhibitors
AVR: Aortic valve replacement
BMI: Body mass index
CI: Confidence interval
CV: Mortality cardiovascular mortality
HR: Hazard ratio
ICD: International classification of diseases
IQR: Interquartile range
LISA: Longitudinal integration database for health insurance and labor market studies
GLP1 RA: Glucagon-like peptide-1 receptor agonists
MESA: Multi-ethnic study of atherosclerosis
MRA: Mineralocorticoid receptor antagonists
NDR: National diabetes register
NPR: National patient register
SD: Standard deviation
SAVR: Surgical aortic valve replacement
SGLT2: Sodium glucose co-transporter-2 inhibitors
TAVR: Transcatheter aortic valve replacement
