# Supplementary material for: Type 2 diabetes is associated with clinical progression of aortic stenosis: a nationwide retrospective study in Sweden
Source: Sci Rep. 2026 Aug 8;16:24550. doi: 10.1038/s41598-026-64970-2 (PMC13452801; doi:10.1038/s41598-026-64970-2)
Supplement: Supplementary file 1 [file 41598_2026_64970_MOESM1_ESM.docx]

Supplementary Table 1: ICD and ATC codes used in the study

|  | ICD9 | ICD10 |
| --- | --- | --- |
| AS | 395A, 424B, 746D | I35.0, I35.2, I06.0, I06.2, Q25.3, Q 23.0 |
| AS of rheumatic or congenital etiology | 395A, 746D | I06.0, I 06.2, Q25.3, Q23.0 |
| Cardiovascular mortality | 390-459 | I 00-I 99 |
| TAVR | 35.05 | FMA10, FMD12, FMD13 |
| SAVR | 3116, 3117, 3074, 3075 | FMD00, FMD 10, FMD20, FMD 30, FMD33, FMD 40, FMD 96 |
| Atrial fibrillation | 427D | I48 |
| Cardiomyopathy | 425A, 425B, 425D, 425E, 425F, 425H, 425W, 277D, | I42, I43, |
| Diabetes | 250 | E10- E14 |
| Coronary heart disease | 410-414 | I20- I25 |
| Myocardial infarction | 410 | I21 |
| Stroke any | 431, 432, 433, 434, 436 | I61-I64 |
| Ischemic stroke | 433, 434 435X, | I63, I64 |
| Hemorrhagic stroke | 431, 432 | I61, I62, |
| Congenital heart disease | 745-747 | Q33.2, Q87.2, Q87.4, Q87.8, Q2, Q874E, Q2, Q92, Q96, Q20-Q24, Q28 |
| Heart failure | 428 | I50.0, I50.1, I50.9, I11.0, I13.0, I13.2 |
| Hypertension | 401-405 | I10- I15 |
| Obesity | 278A, 278B | E65, E66. |
| Valvular disease | 394-396, 424 | I05-I09, I35-I39 |
| Deep venous  thromboembolism | 451B, 451C | I80.1-I80.9 |
| Pulmonary embolism | 415A, 415B, 673C, 639G | I26 |
| Aortic aneurysm/dissection | 441 | I71 |
| Cancer | 140-208 | C00-C96, D00-D09 |
| Renal failure | 584-588, V45B, V56 | N17-N19, I12.0, DR016, DR023, DR056, Z940, |
| COPD / Asthma | 490-493 | J40-J45 |
| Hypercholersterolemi | 272A | E78.0 |
| Hyperlipidemia | 272A, 272B, 272C | E78 |
| Peripheral arterial disease | 440C, 443X | I70.2, I73.9 |
| Endocarditis | 421 | B37.6, I33.0, I33.9, I38, I39.8 |
| Sleep apnea disease | 327.20-327.29 | G47.3 |
| HyperPTH | 252.A | E21.0-E21.3 |
| ACEi/ARB/ARNi |  | C09AA01, C09CA, C09DX04 |
| MRA |  | C03E |
| SGLT2 |  | A10BK |
| Beta-blockers |  | C07AA, C07AG02 |
| Diuretics |  | C03A, C03C, C03B |
| Anticoagulation |  | B01AA, B01AE07, B01AF |
| Oral antidiabetics |  | A10B (except A10BJ) |
| Insulin |  | A10A |
| Glucagon-like peptid-1-receptor analogs |  | A10BJ |
| Statins |  | C10AA |
| Antiplatelet |  | B01AC04, B01AC06, B01AC22, B01AC24. |

AS aortic stenosis, ACEi angiotensin converting enzyme inhibitor, ARB angiotensin receptor blockers, ARNi Angiotensin Receptor-Neprilysin Inhibitors, MRA mineralocorticoid receptor antagonists, SAVR surgical aortic valve replacement, SGLT2 sodium glucose co-transporter-2 inhibitors, TAVR transcatheter aortic valve replacement.

Supplementary table 2- Missing variables in NDR

| **Missing variables in NDR** | **n(%)** |
| --- | --- |
| DM duration | 1857 (11.5) |
| Smoking | 3259 (20.2) |
| Albminuria | 6757 (41.9) |
| GFR | 3438 (21.3) |
| Retinopaty | 10824 (67.2) |
| Systoplic BP | 1902 (11.8) |
| Diastolic BP | 1926 (12.0) |
| BMI | 3837 (23.8) |
| Creatinine | 3438 (21.3) |
| HbA1C | 1509 (3.4) |
| Cholesterol | 5032 (31.2) |
| Physical activity | 7742 (48.0) |

Supplementary Figure 1: Forest plot of factors influencing composite outcome in cases and controls
